# Vaccination in Islamic countries: a narrative review

**DOI:** 10.3389/phrs.2026.1609007

**Published:** 2026-08-04

**Authors:** Antonia-Olivia Roberts, Shahid Jameel

**Affiliations:** 1 University of Oxford Green Templeton College, Oxford, United Kingdom; 2 Oxford Centre for Islamic Studies, Oxford, United Kingdom

**Keywords:** climate change, Islamic countries, vaccine access, vaccine hesitancy, vaccines

## Abstract

Vaccination is a key public health intervention, and Muslims who comprise a quarter of the world’s population, have shown mixed responses to it. Previous research has addressed Islamic perspectives on vaccination but understanding its efficacy from secular and religious points of view across Islamic countries is limited. This narrative review utilised database search (Google Scholar, PubMed, Scopus, SSRN, and Web of Science) followed by curation based on expert knowledge. It offers theological and technological insights, and the potential impacts of climate change on vaccination in Islamic countries. Though Islamic teachings support vaccination, hesitancy results from a complex mixture of sociological and technological barriers that include misinformation, lack of trust, poor living conditions, and vaccine inequity in resource-limited communities. Climate change will act as a threat multiplier, particularly in areas inhabited by over 80 percent of the world’s Muslims. Successful approaches from Islamic countries highlight the role of cultural and religious perspectives, infrastructure, and biological factors on the coverage and efficacy of childhood, adolescent and adult vaccines. The learnings offer strategies to improve vaccination everywhere, but particularly in Muslim populations.

## Introduction

Vaccination is an important public health intervention, which has halved child mortality, saved an estimated 154 million lives in the last 50 years [1, 2], and continues to prevent about 4.4 million deaths annually [3]. Vaccines also reduce poverty by limiting out-of-pocket medical expenditure, increase literacy by keeping children in school, decrease the use of antibiotics, and reduce the impact of pathogen spillovers which may be aggravated by climate change [4]. However, these can only be achieved if vaccines reach large swathes of the population, which relies on efficient healthcare systems and the willingness to be vaccinated. Consequently, successful vaccination campaigns also include cultural sensitivity in their messaging, planning and implementation.

The Organisation of Islamic Cooperation (OIC) includes 57 countries with either a majority or a significant Muslim population [5] that are heterogenous by way of economic, political, cultural and regional diversity. As Muslims constitute about a quarter of the world population, predicted to reach about 30 percent by 2050 [6], understanding their socio-cultural and religious attitudes to vaccination is important. Due to complacency, conflicts, mistrust, and misunderstood religious positions on vaccination, the only countries where polio remains endemic, are both Islamic (Afghanistan and Pakistan). Some recent vaccines are also used less commonly in Islamic countries. The Vaccine Confidence Project surveyed over 280,000 people from 149 countries between 2015 and 2019 [7]. Its findings show declining confidence in vaccines in six countries–Afghanistan, Azerbaijan, Indonesia, Nigeria, Pakistan and Serbia–five of which are OIC member countries.

In this narrative review we provide a broad, interpretive summary of vaccination in Islamic countries and examine it through the lens of religious guidance on health and wellbeing. It will revisit the challenges and barriers to vaccination faced by low-and-middle-income countries (LMICs), particularly under the impacts of climate change, with an emphasis on Muslim societies. With success stories and best practices, we explore how to lower barriers and overcome challenges.

## Overview of vaccination in Islamic countries

Vaccination across Muslim-majority countries is uneven, shaped less by religion than by state capacity, conflict, financing, public trust and health-system reach. The diphtheria-tetanus toxoid-pertussis vaccine (DTP3) involves three doses and is a good indicator of access to vaccination services [8]. The mean of one-year-olds immunised with DTP3 is 83.6 percent for OIC countries and 87.3 percent for others. The rotavirus and pneumococcal vaccines introduced around the year 2000 have an estimated coverage of 78 percent and 80 percent respectively in OIC countries, against the global average of 76 percent and 83 percent [9]. But for the newly developed COVID-19 vaccine, only 50.8 percent of people in OIC countries had received at least one dose by 2022, compared to 64.3 percent globally [10].

**FIGURE 1:**
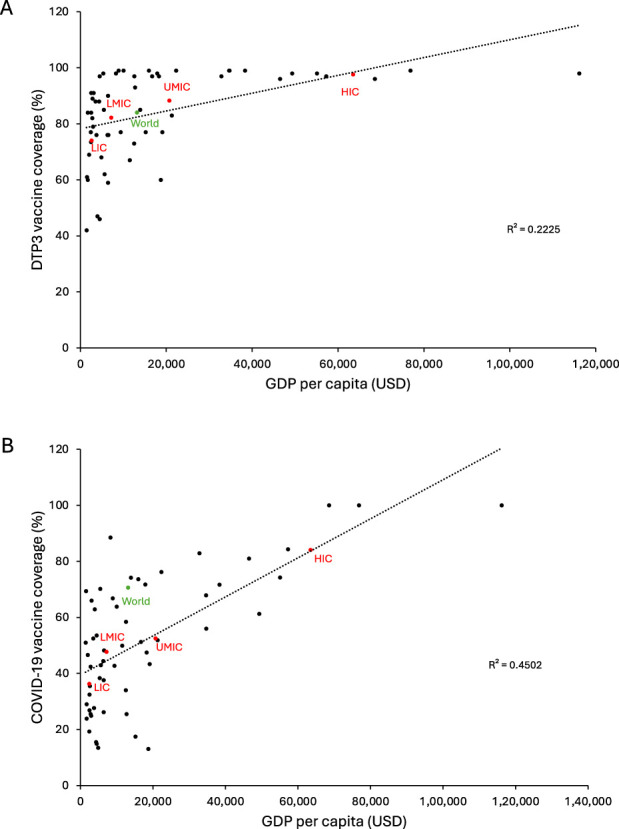
Correlation between a country’s *per capita* GDP and vaccination coverage in Organization of Islamic Cooperation (OIC) member countries are shown for **(A)** Diptheria-Tetanus-Pertusis (DTP3), and **(B)** COVID-19 vaccines. The COVID-19 coverage shows percent of population with one or more doses by 2024. The averages for low-income countries (LIC), lower middle-income countries (LMIC), upper middle-income countries (UMIC), and high-income countries (HIC) are shown in red and the world average in green. The correlation coefficients for vaccination coverage as a function of *per capita* GDP are 0.22 and 0.45 for DTP3 and COVID-19, respectively. The GDP data is from The World Bank (2023); the DTP3 and COVID-19 vaccination coverage is from Our World in Data (2024).

The association between *per capita* GDP of OIC countries and their DTP3 and COVID-19 vaccine coverage is shown in Figure 1. The established DTP3 childhood vaccine has better coverage compared to the adult and emergency use COVID-19 vaccine. Notably, the same countries are found above or below the median for both DTP3 and COVID-19 vaccinations, indicating why robust in-country vaccination infrastructure is important in countering emerging health threats. The new COVID-19 vaccines developed during the pandemic were expensive and in short supply [11]. Understandably, the correlation between vaccine coverage and *per capita* GDP is better for the COVID-19 vaccine compared to the DTP3 vaccine, which is now routinely available to low- and low-and-middle-income countries.

Funding is a key driver of inter-country variation in vaccination rates. In LMICs the government contributes 63.2 percent of vaccination spending, with 30.4 percent coming from development assistance agencies such as the Global Alliance for Vaccines and Immunization (GAVI) [12]. Contributions from the private sector, non-governmental organisations, faith-based organisations and for-profit clinics, vary considerably across settings [13].

## Islamic perspectives on vaccination

### Theological position on vaccination

According to Dar Al-Ifta, an Egyptian Islamic advisory, justiciary and governmental body – “In Islam, the sanctity of life is an inherent right for every human being because life is a God-given gift and a manifestation of His Divine grace” [14]. This line of reasoning, which also follows from earlier Abrahamic traditions, is used to interpret theological support for vaccination in Islam. For example, when asked if it was a sin to not seek treatment, the Prophet Mohammad (peace be upon him) is reported as saying, “Seek treatment, O slaves of Allah! For Allah does not create any disease but He also creates with it the cure, except for old age” [15]. There is a consensus among Islamic scholars that seeking vaccination is a religious duty because not doing so may lead to transmission of the disease to others and affect the community’s wellbeing [16].

### Vaccine concerns among Muslims

Despite clear theological guidance, misconceptions related to vaccines exist among some Muslims. A survey in Guinea found that 54 percent of common people and 20 percent of religious leaders considered vaccination to be prohibited during the month of Ramadan [17]. However, several religious authorities and organisations, including the President of the General Presidency for the Affairs of the two Holy Mosques in Saudi Arabia have issued statements that receiving a vaccine does not invalidate the Ramadan fast [16].

Another common concern is that vaccine preparations may contain *haram* (or prohibited) ingredients, such as the enzyme trypsin, which is isolated from pig pancreas and is used in the production process for some vaccines. Even when trypsin is used, the World Health Organization (WHO) and European Medicines Agency certify it to be absent from the vaccine product [18]. In 2018, Indonesia’s Central Ulama Council issued a *fatwa* (opinion) declaring that the measles-rubella vaccine had porcine ingredients, but vaccination was still permissible due to its public health benefits [19]. Such mixed messaging confused local clerics and parents, and vaccination rates declined to 8 percent in some provinces [20]. The WHO and religious leaders have used the principle of *istihala*–that chemical reactions lead to loss of the original form–to declare such vaccines to be *halal* (permissible). Recombinant trypsin produced in bacteria or yeasts is now increasingly being used for vaccine and biopharmaceutical manufacturing, negating this concern [21, 22].

While core Islamic concepts support vaccination, the reasons for hesitancy are complex and include social and religious beliefs [16]. In the USA, Muslims showed a higher intent to be vaccinated than the general population, and in Saudi Arabia vaccine hesitancy decreased with increasing education and income [16]. Misinformation and low trust in government contribute to vaccine hesitancy everywhere, including among Muslims.

## Challenges and barriers to vaccination

Vaccination faces both technological and sociological barriers. The former includes vaccine research and development, its production, distribution, and real-world effectiveness. The latter include reduced acceptance due to poor understanding of vaccine science and negative social media messaging, including false claims of links to autism and injury.

### Production of vaccines

**FIGURE 2:**
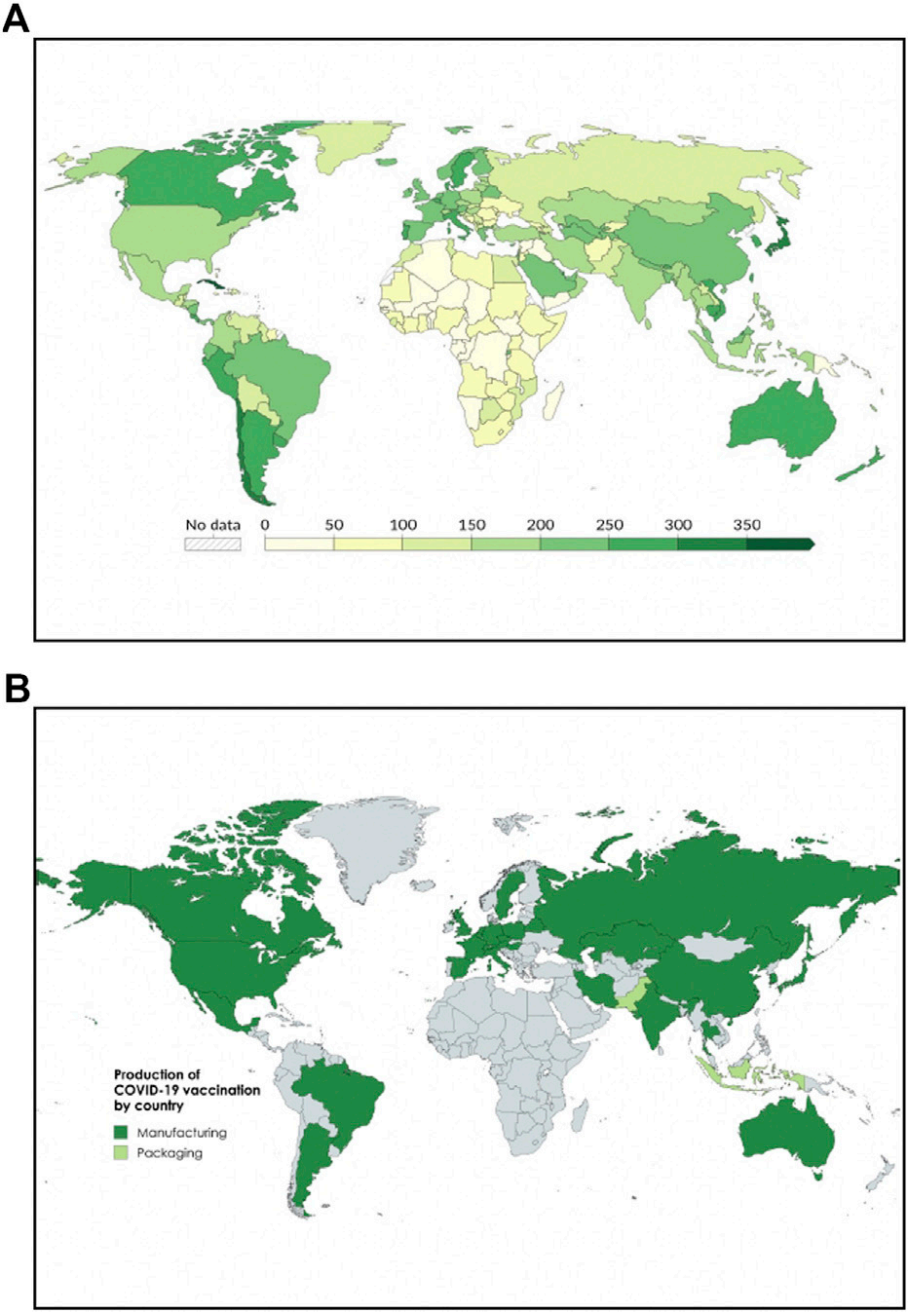
Global COVID-19 vaccination rates correlate with vaccine manufacturing sites. **(A)** Total COVID-19 vaccine doses administered per 100 people by August 2024. **(B)** Countries with COVID-19 vaccine manufacturing and packaging facilities. Data sources: Our World in Data, and https://launchandscalefaster.org/covid-19/vaccinemanufacturing.

Vaccine production is a natural monopoly since scale is needed to ensure affordability, but this reduces equity, especially for new vaccines and in outbreak situations. The production of COVID-19 vaccines was restricted to the US, Europe, China and India–where more than ninety percent of all doses were manufactured [23]. Large parts of the world, including most of Africa, Asia and Latin America, did not produce any of these vaccines. Africa produces less than one percent of its routine vaccines, and produced no COVID-19 vaccines, which correlates with low vaccine uptake across the continent (Figure 2).

Very few vaccine manufacturing sites exist in LMICs and Islamic countries. Iran, Kazakhstan and UAE were the only OIC countries to manufacture any type of a COVID-19 vaccine during the pandemic. Indonesia has good infrastructure for producing polio and other childhood vaccines and has set up a partnership for future pandemic vaccines [24]. Pakistan purchased a Chinese COVID-19 vaccine in bulk and repackaged it for domestic use [25]. However, none of these countries produced sufficient doses to even fulfil domestic requirements [26]. Iran (population 91 million) produced 15 million doses [26] and used over 155 million doses [27]. Kazakhstan (population 20.5 million) produced 9.6 million doses [26] and used 26 million doses [28]. Other OIC countries and LMICs imported COVID-19 vaccines when supplies were limited. Unsurprisingly, a country’s *per capita* GDP correlated well with its COVID-19 vaccine uptake [10, 29], except for India, which has invested in vaccine production for domestic use and export.

### Vaccine supply chains

Getting vaccines to the populations that need them is critical, but areas under conflict present a significant challenge. Four of the six countries with less than 50 percent DTP3 coverage are affected by conflict, and three of these are OIC member states (Guinea, Yemen and Somalia) [3, 30]. Conflict zones also have compromised health systems, but vaccination programmes are particularly vulnerable as their impact is not immediately visible. The destruction of hospitals and healthcare facilities, collapsed sanitation systems, overcrowded refugee camps, and the lack of access to clean water in Gaza, brought back wild type poliovirus to this region in 2024 since its polio-free status in 1999 [31]. A ceasefire enabled three rounds of polio vaccination campaigns between September 2024 and February 2025 to urgently address the problem [32].

Vaccine wastage is yet another challenge. The US Food and Drug Administration has approved 98 vaccines, of which about 70 percent are liquids stored at 2 °C–8 °C with expiration dates ranging from 4 months to 3 years [33]. About 20 percent of the vaccines are freeze-dried powders stored at 2 °C–8 °C, and the rest are frozen liquids. If the frozen vaccines are thawed, the liquid ones inadvertently frozen, or the lyophilized powders not used within a few hours of rehydration, the vaccines are discarded. Nearly 50 percent of vaccines are wasted globally due to improper cold chain [34] and availability in multi-dose vials [35].

Cold chain also presents a significant challenge for LMICs with unstable power supplies [36]. Bad roads and hard to reach areas make it even more difficult to deliver vaccines to needy populations. But these problems have also attracted novel approaches and disruptive innovation. Solar refrigerators that connect directly to solar panels, instead of relying on an unstable power grid or backup batteries, can keep vaccines at the right temperature [37]. In hard to reach areas, biker teams have distributed polio drops to nomadic settlements in Afghanistan [38], and drones are being used in Africa to deliver vaccines, essential drugs and blood supplies to remote areas [39]. Administering vaccines requires good infrastructure including clinics, healthcare workers and documentation systems. Several studies highlight logistical barriers that deter families that are otherwise accepting of vaccines [30, 40]. One survey of families in Pakistan where children had not received the polio vaccine found the absence of visits by vaccination teams to be the most common reason for it [41]. The density of vaccination workers correlates with immunisation coverage for several other vaccines [42].

### Vaccine acceptance

Vaccine refusal among Muslims is another barrier, a common concern being the perceived *haram* status of certain vaccines [41]. Conspiracy theories and the belief that polio vaccines will lead to infertility in Muslim children have reduced its acceptance in Nigeria [43] and Pakistan [44]. In 2007, Sudan’s Muslim Brotherhood issued a *fatwa* against all vaccines on the unfounded claim of being the conspiracy of Jews and Freemasons [16]. Often these theories arise from a lack of trust in the state’s intentions [30]. Given the legacy of disease research on vulnerable populations, and the use of vaccination programmes to achieve political goals, these concerns cannot be ignored. The Tuskegee Syphilis Study on black men, and the deliberate infection of intellectually challenged children at Willowbrook State School to understand the aetiology of viral hepatitis, are examples of the painful legacy of vaccine development [45, 46]. The US Central Intelligence Agency’s fake vaccination programme in 2011 to track Osama bin Laden is another act that reduced trust and set back polio eradication in Pakistan and Afghanistan [47]. In January 2021, when COVID-19 cases were rapidly rising in Iran [48], its leaders ruled out importing vaccines from either USA or UK due to their mistrust of the West. A 2023 modelling study estimated that earlier vaccination of vulnerable groups in Iran could have prevented over 75,000 deaths during the first two years of COVID-19 [49].

### Vaccine effectiveness

The immune response to vaccines is variable, with some oral vaccines being less effective in resource poor settings [50, 51]. The reasons are complex, including malnourishment and enteric infections, which impair T-cell memory responses [52]. Gastrointestinal infections worsen a child’s ability to absorb nutrients, leading to a vicious cycle of undernourishment, immunosuppression and increased susceptibility to vaccine preventable diseases [53]. *Campylobacter* and enterovirus infections decrease the protection offered by oral vaccines against polio and rotavirus diarrhoea [54, 55]. Such enteric infections are more common in areas without access to safe water and disproportionately affect low-income countries. Islamic countries show slightly more under-nourishment (13 percent) compared to others (10 percent). They also have higher mortality (18.2 per 100,000 people) that is attributed to unsafe water, compared to others (10.1 per 100,000 people) [56]. As reduced vaccine effectiveness increases hesitancy in societies that need them the most, nutrition-focussed interventions and improvements to personal and community hygiene are as important but have received insufficient attention.

### Digital misinformation

Digital misinformation circulates in many Islamic countries, just as it does elsewhere. Common narratives including non-halal ingredients, links to infertility, foreign conspiracies, and concealment of dangerous side effects have appeared against polio, measles and COVID-19 vaccines. Research shows that social media platforms have enabled rapid dissemination of vaccine misinformation globally, including in low- and middle-income Muslim-majority countries. The most effective approaches to counter these include engagement with trusted religious leaders and public endorsements from Islamic scholars; transparency of vaccine ingredients and halal certification; community-based instead of top-down communications; monitoring and rapid response to misinformation; and building trust in institutions [57].

## Climate change and vaccination

There is now increasing attention to the impacts of climate change on human health. About 60 percent of human pathogens are estimated to be aggravated by climate hazards, making preparedness, including the development of new vaccines, a priority [58, 59]. This is especially relevant for Muslim societies since over 80 percent of the world’s Muslims live in areas most prone to climate change.

### Impact on disease transmission

Environmental threats are projected to cause animal and human migration into new areas, resulting in novel interactions between mammalian species, and an estimated 4,000 new episodes of cross-species virus transmission [60]. Most virus-sharing events are predicted for the Indian subcontinent, Sahel, and Southeast Asia. These areas also have large Muslim populations.

Changing pathogen distribution will also impact regional vaccine requirements. Insect vectors will multiply faster because of rising temperatures and longer monsoons [61], exacerbating vector-borne diseases, an example being the worst ever dengue outbreak in Bangladesh in 2023 [58]. Malaria transmission has already gone up in two- to ten-year-olds in Sub-Saharan Africa [62]. *Plasmodium falciparum*, the most dangerous form of malaria, is transmitted by the *Anopheles gambiae* mosquito that propagates best at 25 °C. With rising temperatures its distribution might shift from Sub-Saharan to Southern or Eastern Africa [61]. The *Aedes aegypti* mosquito transmits several viruses including the dengue virus, multiplies best at 29 °C, and its global spread is increasing. Though new malaria vaccines have recently become available, endemic countries cannot afford these at about $10 per child [63].

It is estimated that by 2050 over 5 billion people will be exposed to at least 1 month of extreme heat annually, with several countries in Asia, the Middle East and Africa having already crossed that threshold [64]. Erratic water patterns will damage sanitation systems and push people to use unsafe water, increasing the transmission of waterborne diseases [61]. Extreme heat is also associated with increased infectious diarrhoea caused by bacteria such as *Salmonella* and *Shigella*, which manipulate the host’s metabolism and lower immunity [65]. Heat and changing vector patterns will necessitate changes in national immunisation programmes, and emergency vaccination in the affected areas.

### Impact on vaccine delivery

Extreme weather events such as floods reduce access, especially for people in rural and hard to reach places [59]. Increasing heat reduces attendance at health facilities and affects routine immunisation services due to the breakdown of cold chain equipment. Climate change will cause population displacement, making it harder for immunisation services to track people and provide routine vaccinations [59]. Further, the areas that climate refugees are displaced into may not have adequate health infrastructure to provide routine care to rapidly increasing populations [58].

### Impact on vaccine effectiveness

Climate change is predicted to reduce crop yields, increasing cereal prices and worsening malnutrition, with stunting expected to rise by 23 percent in Sub-Saharan Africa and 62 percent in South Asia [66]. These regions are also home to about 50 percent of the world’s Muslims, who are largely poor. Reduced access to food and other resources will cause population displacement, with people moving into informal housing with poor sanitation, further exposing them to enteric infections [59, 66]. Climate-related extreme weather increases air pollution, reduces biodiversity, and causes psychological stress, all of which lower immunity [66, 67]. Since undernutrition and stress decrease vaccine-derived immunity and increase susceptibility to infections, it would be even more important to expand immunisation programmes to underserved populations.

## Success stories and best practices

To improve vaccination, it is instructive to learn from successful examples from the Muslim world. We highlight smallpox and polio eradication programmes, and Islamic countries that have vaccinated their populations beyond their socioeconomic status and the prevailing religious environment.

### Smallpox eradication

For centuries smallpox remained a major cause of morbidity and mortality, killing an estimated 300–500 million people in just the 20th century [68]. Though modern vaccination is attributed to Edward Jenner, who in 1796 used cowpox to protect against smallpox, variolation was widely practised in the Orient and was brought to England in the early 18th century [69].

Within the Islamic world, the contributions of the Ottomans to smallpox control are particularly noteworthy [70]. Sultan Abdulmejid I (ruled 1839–1861) built medical research institutions, provided free vaccination and published the treatise *Menafiu’l-etfal* (*Benefits to Children*) to dispel common myths. Similar efforts were undertaken by Muhammad Ali (ruled 1805–1849) in Egypt and Crete [70]. In 1967, the World Health Organization launched an intensified plan to eliminate smallpox, culminating in its eradication in 1980 – the only infectious disease affecting humans to be eradicated [71]. The lack of animal reservoirs for smallpox, the existence of a heat-stable single-dose vaccine, intensive surveillance to identify new cases, and ring vaccination of contacts were important for its eradication [72, 73]. Proper training and supervision of minimally educated volunteers [72], good tools and logistics and gaining the trust of key stakeholders, are important lessons from smallpox eradication.

### Towards polio eradication

The global vaccination campaign against poliovirus is another success, with the wildtype virus currently endemic only in Afghanistan and Pakistan [38]. Efforts in these countries have successfully reduced vaccine hesitancy by involving influential people and religious leaders [30]. In areas with high rates of vaccine refusal, vaccinators carry *fatwa* pamphlets with endorsements from Islamic scholars [74] and religious leaders have delivered pro-vaccine sermons [30]. The female mobiliser vaccine network in Afghanistan, which includes local women, has helped vaccinate hard-to-reach children through health education sessions, access to mothers, and providing vaccines in their local communities [75].

Many under-immunised children are also under-nourished, reinforcing the importance of a public health strategy that integrates nutrition and immunisation services. For example, vitamin A deficiency being widespread, it is given with the oral polio vaccine on national immunisation days [76]. Free school meals are available to 41 percent of primary school children worldwide, but to only 18 percent of children in low-income countries [77], which deserves urgent attention.

### Vaccination in Bangladesh

Bangladesh’s routine vaccination rates are better than countries of comparable *per capita* GDP – 98 percent DTP3 coverage for one-year-olds, and 89 percent of the population received at least one dose of a COVID-19 vaccine [10]. Public opinion in Bangladesh favours vaccination, with 99 percent agreeing that vaccines are safe [78]. During the pandemic, social media updates increased public awareness and vaccine demand [79]. Bangladesh received NGO support for over half of its routine immunisation services [80] and had the most COVID-19 vaccine donations from COVAX, China and India [79]. Reducing out-of-pocket expenditure, and structural improvements such as a digital District Health Information System that records all vaccinations, made it easier to identify under-performing areas for targeted action, and increased the efficiency of vaccine administration [80]. The lessons from Bangladesh include the importance of public understanding of vaccination, the role of NGOs, and effective technologies supporting national immunisation programmes.

### Cervical cancer vaccination in Malaysia and Uzbekistan

Cervical cancer, caused by the human papilloma virus (HPV), is the second leading cause of cancer deaths among women in less developed countries [81]. Islamic countries have lower rates of HPV infection due to conservative sexual behaviours, but poor screening makes it difficult to quantify prevalence and changing trends [81]. Three OIC countries–Mozambique, The Comoros and Guyana–have a particularly high burden of cervical cancer [82]. The HPV vaccine given during adolescence is routinely used in only 20 of 57 OIC countries compared to around three-quarters of other countries [83]. But OIC countries that use the HPV vaccine have population coverage like other countries [84], suggesting that a vaccine is accepted once it is included in the programme. Fewer Muslim countries mandating its use could be due to a reluctance to discuss sexual health, or the perception that consent to vaccinate encourages sexual activity [85].

Malaysia follows a school-based HPV programme that has high parental consent and 95 percent vaccination of eligible schoolgirls [86]. In Uzbekistan 94 percent of 12–14-year-old girls have received the first dose of an HPV vaccine [87]. Such high rates are due to clear communication with messaging focused on maternal health and protecting motherhood rather sexual health [87]. Good communications, data transparency, and promptly addressing misinformation are key to these successes. Other OIC countries such as Bangladesh, Nigeria and Togo are following this successful model [88].

### The GCC countries

The Gulf Cooperation Council countries had some of the highest COVID-19 vaccine uptake globally due to strong government leadership (vaccine procurement, free vaccination), good digital infrastructure, and healthcare access. Studies in the region show that while COVID-19 vaccine acceptance was high due to an immediate threat perception, HPV vaccination faces challenges related to awareness, perceived risk, and cultural sensitivities. Other than Abu Dhabi, which has >95% HPV vaccine uptake through school-based programmes, the rest of the region shows <10% uptake [89]. Studies emphasize the importance of regional collaboration and long-term self-sufficiency through sustainable financing, local manufacturing to improve vaccine access and equitable outcomes across the Middle East [90].

## Strategies for improving vaccination

There is a need to learn from success stories, include new strategies into existing programmes, and to be particularly mindful of adult vaccination to counter climate-related exacerbations of infectious diseases. Access to emergency vaccines remains a key barrier, especially for low-income countries and LMICs in Africa, Asia and Latin America.

### Vaccine production

Vaccine manufacturing should be expanded globally to include more countries and regions. A 2023 study identified 43 countries, including 11 OIC member states, with the capacity to produce WHO pre-qualified vaccines [91]. Two OIC countries–Tunisia and Azerbaijan–are particularly strategic due to existing industrial capacity and small population size, allowing them to provide for their population and export to other countries [91]. Several other Islamic countries such as Kuwait, Oman, Qatar, the UAE and Brunei Darussalam have skilled manpower and sovereign wealth funds that can leverage investments for social good, and serve as good regional vaccine production hubs.

Africa’s population is expected to grow rapidly from 1.5 billion in 2024 to 3.8 billion by 2100 [92], of which 40 percent are Muslim. Africa also bears a disproportionate burden of infectious diseases with morbidity and mortality that hinders sustainable development. The continent’s experience with HIV/AIDS research could guide the development of new vaccines in Africa [93]. Vaccine R&D, innovation and intellectual property are presently concentrated largely in the global North. It will take political will, sustainable regional markets, and a strong regulatory environment to overcome these barriers [94]. The WHO Pandemic Treaty may help improve access to technology and improve vaccine equity [95].

### Delivery of vaccines

Vaccine access is limited in remote areas with bad roads. Drones are being used to deliver vaccines, essential drugs, and blood supplies in these areas, with lower emissions compared to road transport [58, 96, 97]. Changes in vaccine provision, such as using polio immunisation days to administer other routine immunisations allow for the sharing of workers, facilities, and knowledge to improve all vaccinations. Providing nutritional support in schools, increases vaccine effectiveness and improves coverage and learning outcomes by reducing sick days and keeping children in school [76].

Making access more convenient improves vaccine uptake. India’s highly successful Pulse Polio Immunisation Programme uses advertising, neighbourhood camps and mobile teams to reach millions of children close to where they live [98]. In Uganda, accessibility was improved by advertising temporary National Immunisation Days within walking distance from home [30]. As global warming makes extreme heat more widespread, vaccination services will also have to adapt. In India, immunisation services are opening earlier in the day when it is cooler [59].

### Vaccine acceptance

Vaccination is positively reinforced through culturally sensitive and community-centred campaigns that address people’s religious, cultural, and socioeconomic concerns. For Islamic countries, this includes the involvement of influential community and religious leaders in planning and implementation, and to encourage the devout to accept vaccines [30]. When endorsed by faith leaders, devout Muslims consider vaccination a religious obligation to promote health and preserve life and get vaccinated while observing the Ramadan fast. Communication plans that emphasise the safety, efficacy, and importance of vaccination in simple language dispel concerns and misinformation. Targeted outreach programs analogous to the female mobiliser networks in Afghanistan and school meetings in Uzbekistan can be used to address vaccine disparities within a region [75, 87].

## Conclusion

Universal childhood immunisation rates in Islamic countries are as good as elsewhere, but some adolescent and adult emergency vaccines show reduced coverage. Despite the success of global polio eradication, the only two countries where wildtype poliovirus can still be found have predominantly Muslim populations. This results from misinformation and misrepresentation of theology. Technological and socio-economic factors such as the paucity of countries that can produce and deliver enough vaccines also contribute to reduced coverage.

Climate change will act as a threat multiplier, magnifying the difficulties in vaccinating populations in low income or hard-to-reach areas. It will reduce vaccine effectiveness and shape the types of vaccines required in the future. These problems are already apparent, and urgent action is needed to adapt and improve existing vaccination programmes.

To reduce inequity and improve access, vaccine production should be expanded to include a wider range of countries across different regions, with a focus on low and low-middle income countries. Novel approaches to vaccine delivery should be explored, with the use of emerging technologies or joining up vaccination with other interventions to provide holistic healthcare. Consultations with locally respected figures, and in many Muslim populations the endorsement of religious leaders is highly beneficial. Whilst the threats to vaccination in Islamic countries are multiple, progress has been made already, with millions of lives saved. Reflecting on the experience gained from the past and combining it with new technologies is a good recipe to prepare for emerging disease threats.

Being a narrative review, our work is limited by a lack of reproducible methodology for inclusion and exclusion, and may therefore be susceptible to selection bias, publication bias, and subjective interpretation.
